# Morbidity and mortality associated with fracture of the sternum due to blunt trauma, by fracture type and location

**DOI:** 10.1590/0100-3984.2021.0074

**Published:** 2022

**Authors:** Sadullah Şimşek, Cihan Akgül Özmen, Serdar Onat

**Affiliations:** 1 Dicle University, Medical School, Diyarbakır, Turkey.

**Keywords:** Sternum/injuries, Fractures, bone/mortality, Wounds and injuries, Esterno/lesões, Fraturas ósseas/mortalidade, Ferimentos e lesões

## Abstract

**Objective:**

To examine the relationship that the types and locations of fractures of the sternum have with mortality and morbidity.

**Materials and Methods:**

We analyzed the records of 115 patients diagnosed with fracture of the sternum, due to blunt trauma, between 2007 and 2018. Records of computed tomography studies were obtained from the radiology archive of a tertiary teaching hospital. The type of fracture was classified as linear, displaced, or comminuted, whereas the fracture location was classified as the manubrium, body, or xiphoid process.

**Results:**

A total of 108 patients were included in the study. Of those patients, 92 (85.2%) were male and 16 (14.8%) were female. The etiology was a traffic accident in 72 cases (62.6%) and a fall from height in 36 (31.3%). The mean age was 42.1 ± 17.7 years for males and 53.9 ± 20.0 years for females. The mortality rate was 11.1%. Among the 12 deceased patients, the mean age was 44.4 ± 18.3 years. The fracture was located exclusively in the manubrium in 64 patients (59.3%), exclusively in the body of the sternum in 41 (38.0%), and in both locations in three (2.7%), whereas none were located in the xiphoid process. Morbidity rates were higher in the patients with fractures of the manubrium than in those with fractures of the body of the sternum, as was the incidence of accompanying bone fractures and organ injuries. The fracture was linear in 44 patients (40.7%), displaced in 62 (57.4%), and comminuted in 30 (27.8%). The mortality was significantly higher for comminuted fractures than for the other fracture types (*p* = 0.045; β = 4.40).

**Conclusion:**

Fracture of the manubrium can be indicative of the severity of trauma and has a poor prognosis.

## Abstract (pt)

**Objetivo:**

Examinar a relação entre o tipo e a localização da fratura de esterno com mortalidade e morbidade.

**Materiais e Métodos:**

Foram analisados os prontuários de 115 pacientes com diagnóstico de fratura de esterno por trauma contuso entre 2007 e 2018. Os registros de estudos de tomografia computadorizada foram obtidos do arquivo de radiologia de um hospital universitário terciário. O tipo de fratura foi classificado como linear, deslocado ou cominutivo, enquanto o local da fratura foi classificado como no manúbrio, no corpo ou no processo xifoide.

**Resultados:**

Cento e oito pacientes foram incluídos no estudo. Desses, 92 (85,2%) eram do sexo masculino e 16 (14,8%) eram do sexo feminino. As causas foram acidente de trânsito em 72 casos (62,6%) e queda de altura em 36 (31,3%). A média de idade foi de 42,1 ± 17,7 anos para os homens e de 53,9 ± 20,0 anos para as mulheres. A taxa de mortalidade foi de 11,1%. Entre os 12 pacientes que faleceram, a média de idade foi de 44,4 ± 18,3 anos. A fratura localizou-se apenas no manúbrio em 64 pacientes (59,3%), somente no corpo do esterno em 41 (38,0%) e em ambas as localizações em três pacientes (2,7%), e nenhuma ocorreu no apêndice xifoide. As taxas de morbidade foram maiores nos pacientes com fraturas no manúbrio do que nos com fraturas no corpo do esterno, assim como a incidência de fraturas ósseas e lesões de órgãos adjacentes. A fratura foi linear em 44 pacientes (40,7%), deslocada em 62 (57,4%) e cominutiva em 30 (27,8%). A mortalidade foi significativamente maior nos casos de fraturas cominutivas do que em outros tipos de fratura (*p* = 0,045; β = 4,40).

**Conclusão:**

A fratura do manúbrio pode ser um indicativo da gravidade do trauma e de mau prognóstico.

## INTRODUCTION

Although fractures of the sternum account for only a small proportion of the injuries observed in patients admitted to the emergency room due to thoracic trauma, their diagnosis and follow-up are important because they can be indicative of cardiac injuries^(1)^. The most common causes of fractures of the sternum are traffic accidents and other types of blunt trauma to the abdomen or thorax. Fractures of the sternum are currently seen in 4% of all traffic accidents and in 3-8% of all cases of blunt abdominal trauma^(2,3)^.

In recent years, technological developments and higher vehicle speeds have increased the traffic accident rate. Seat belts are seen as playing an important role in that increase. Andrews et al.^(4)^ identified fracture of the sternum as a feature of seat belt syndrome. Porter et al.^(1)^ found that fractures of the sternum are 0.7-4.0% more common among trauma patients who were secured with seat belts than among those who were not.

Most fractures of the sternum (> 95%) are treated conservatively, because an isolated fracture of the sternum is seen as a relatively benign injury. Conservative treatment options include analgesia, rest, and passive reduction of the displacement if necessary. Surgical treatment may be required in cases of displaced fractures. Surgical fixation is indicated in case of fracture instability, displacement, or non-union^(5)^. Mortality rates associated with fracture of the sternum are increasing due to an increase in the frequency of accompanying findings. The associated thoracic injuries commonly encountered include vertebral fracture, rib fracture, fracture of the clavicle, fracture of the scapula, pulmonary contusion/laceration, hemopneumothorax, and mediastinal injury.

Although fractures of the sternum are described in the literature as signs of other systemic injuries, there have been, to our knowledge, no studies supporting that fact. Therefore, the aim of this study was to examine the correlations that the type and location of fracture of the sternum show with accompanying systemic findings.

## MATERIALS AND METHODS

This was a retrospective study of the records of 115 patients diagnosed with traumatic fracture of the sternum between 2007 and 2018 at a tertiary teaching hospital. Computed tomography (CT) records were obtained from the radiology archive of the hospital. Seven (6.1%) of the cases were excluded because the patients were gunshot wound victims or had suffered another type of penetrating trauma. Age, sex, and mortality data were recorded. The type of fracture was categorized as linear, displaced, or comminuted, whereas the fracture location was categorized as the manubrium, sternal body, or xiphoid process. All patients underwent imaging examinations to identify any concomitant rib fractures, vertebral body fractures (with or without spinal injury), pneumothorax, hemothorax, pneumomediastinum, pulmonary contusions or lacerations, intracranial hemorrhage, intra-abdominal injuries, or vascular injuries. The CT scans had been acquired in a 256-slice multidetector scanner (Activion 256; Toshiba, Tokyo, Japan). All of the scans were examined by the same radiologists, in the volume rendering mode, as well as in the axial, coronal, and sagittal planes.

The correlations between categorical variables were examined with the chi-square test. After thus examining the variables associated with mortality, we performed a logistic regression analysis of those that were found to be significant. The correlations that the locations and types of fractures of the sternum showed with mortality and other bone fractures or systemic injuries were also tested, with univariate and multivariate analyses. The study was approved by the local research ethics committee.

## RESULTS

**Figure 1:**
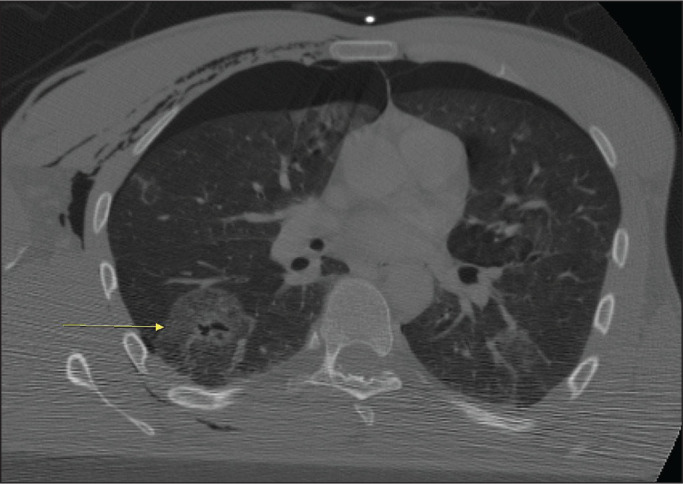
CT scan showing pulmonary contusion (arrow) in a patient with traumatic fracture of the sternum. Note also the pneumothorax and subcutaneous emphysema.

**Figure 2:**
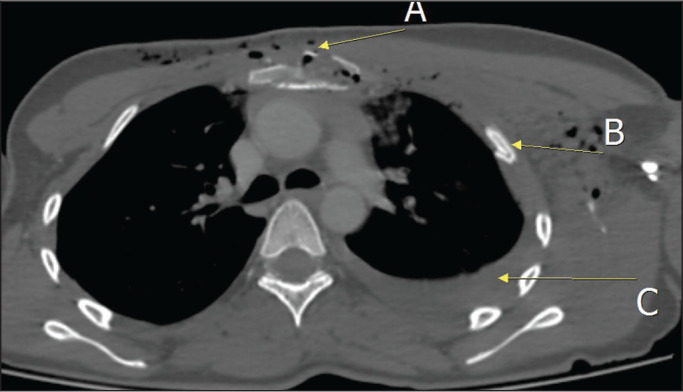
CT scan of a patient who suffered a traffic accident, showing the comminuted-displaced fracture lines in the manubrium (A), rib fractures (B), and hemothorax (C) on the left.

**Figure 3:**
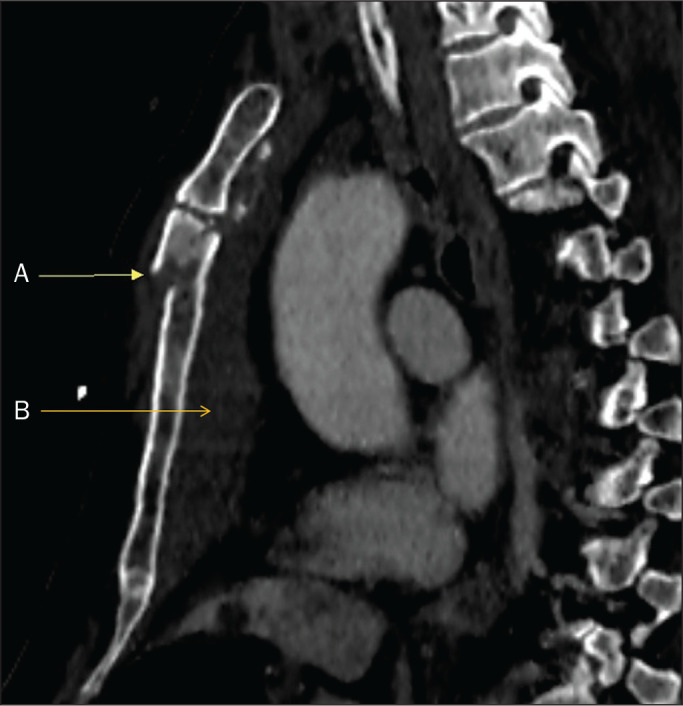
CT scan showing a displaced fracture (A) and retrosternal hematoma (B) in the body of the sternum

A total of 108 patients were included in the study. Of those patients, 92 (85.2%) were male and 16 (14.8%) were female. The etiology of the fracture of the sternum was a traffic accident in 72 cases (62.6%) and a fall from height in 36 (31.3%). A summary of the findings is provided in Table 1. The mean age was 42.1 ± 17.7 years for men and 53.9 ± 20.0 years for women. Patient ages ranged from 11 years to 87 years. The mortality rate was 11.1%. Among the 12 deceased patients, the mean age was 44.4 ± 18.3 years. A summary of the findings according to the fracture location is provided in Table 2. The fracture was located exclusively in the manubrium in 64 patients (59.3%), exclusively in the body of the sternum in 41 (38.0%), and in both locations in three patients (2.7%), whereas none of the fractures were in the xiphoid process. Figures 1, 2, and 3 show aspects of sternal fractures in the manubrium and sternal body. The fracture was linear in 44 (40.7%) of the patients, displaced in 62 (57.4%), and comminuted in 30 (27.8%). According to the logistic regression analysis (Table 3), the location of the fracture of the sternum (manubrium or body) was not associated with mortality, although mortality was significantly higher in patients with comminuted fractures than in those with other types of fractures [odds ratio (OR) = 4.40; *p* = 0.045]. When all bone fractures and other systemic injuries were included in the univariate analysis, comminuted fracture of the sternum, abdominal organ injury, and intracranial injury were found to be significant risk factors for mortality (*p* = 0.012, 0.003, and 0.014, respectively). However, only intracranial injury retained that significance in the multivariate analysis (OR = 9.1; *p* = 0.010).

**Table 1:** Characteristics of and findings in trauma patients with fractures of the sternum.

Variable	(N = 108)
Gender, n (%)	
Female	16 (14.8)
Male	92 (85.2)
Sternal fracture location, n (%)	
Manubrium	67 (62.0)
Body	43 (39.8)
Sternal fracture type, n (%)	
Linear	44 (40.7)
Displaced	62 (57.4)
Comminuted	30 (27.8)
Concomitant fractures, n (%)	
Rib	78 (72.2)
Scapula	17 (15.7)
Clavicle	11 (10.2)
Thoracic vertebra	35 (32.4)
Concomitant organ injury, n (%)	8 (7.4)
Concomitant multi-organ injury, n (%)	2 (1.9)
Concomitant intra-abdominal injury, n (%)	
Myocardial contusion	27 (25.0)
Pericardial hematoma	64 (59.3)
Pneumothorax	58 (53.7)
Hemothorax	56 (51.9)
Pneumomediastinum	12 (11.1)
Pulmonary contusion	58 (53.7)
Pulmonary lacerations	17 (15.7)
Concomitant head injury, n (%)	
Intracranial hemorrhage	20 (18.5)
Subarachnoid	19 (17.6)
Epidural (hematoma)	1 (0.9)
Subdural (hematoma)	7 (6.5)
Parenchymal	8 (7.4)
Death, n (%)	12 (11.1)

**Table 2:** Distribution of the findings by the location of the sternal fractures.

Variable	Location of the sternal fracture*		*P* ^†^
	Manubrium (n = 64)	Body (n = 41)	
Gender, n (%)			0.211
Female	12 (18.8)	4 (9.8)	
Male	52 (81.3)	37 (90.2)	
Rib fracture, n (%)	52 (81.3)	25 (61.0)	0.022
Fracture of the scapula, n (%)	13 (20.3)	4 (9.8)	0.152
Fracture of the clavicle, n (%)	6 (9.4)	5 (12.2)	0.645
Thoracic vertebral fracture, n (%)	18 (28.1)	16 (39.0)	0.171
Myocardial contusion, n (%)	14 (21.9)	13 (31.7)	0.261
Pericardial hematoma, n (%)	35 (54.7)	28 (68.3)	0.165
Pneumothorax, n (%)	41 (64.1)	15 (36.6)	0.006
Hemothorax, n (%)	37 (57.8)	16 (39.0)	0.060
Pneumomediastinum, n (%)	12 (18.8)	0 (0)	0.003
Pulmonary contusion, n (%)	37 (57.8)	19 (46.3)	0.250
Pulmonary lacerations, n (%)	13 (20.3)	4 (9.8)	0.152
Intracranial hemorrhage, n (%)	12 (18.8)	8 (19.5)	0.923
Abdominal organ injury, n (%)	4 (6.3)	3 (7.3)	0.831
Death, n (%)	7 (10.9)	4 (9.8)	0.560

* Three patients (2.7%) had fractures of the manubrium and sternal body, and the analysis was therefore based on the 105 remaining patients.

† Chi-square test.

**Table 3:** Logistic regression analysis of mortality, by the location and type of fracture of the sternum.

Variable	P	OR	95% CI
Fracture location			
Manubrium	0.502	0.398	0.027-5.856
Corpus	0.502	0.398	0.027-5.856
Fracture type			
Linear	0.999	0.000	—
Displaced	0.999	0.000	—
Comminuted	0.045	0.227	0.053-0.966

CI, confidence interval.

Among the 108 patients with fracture of the sternum, the injury was accompanied by rib fracture in 78 (72.2%), thoracic vertebral fracture in 35 (32.4%), fracture of the scapula in 17 (15.7%), and fracture of the clavicle in 11 (10.2%). None of those additional fractures were found to be associated with mortality in the logistic regression (*p* = 0.449, 0.441, 0.552, and 0.852, respectively).

**Figure 4:**
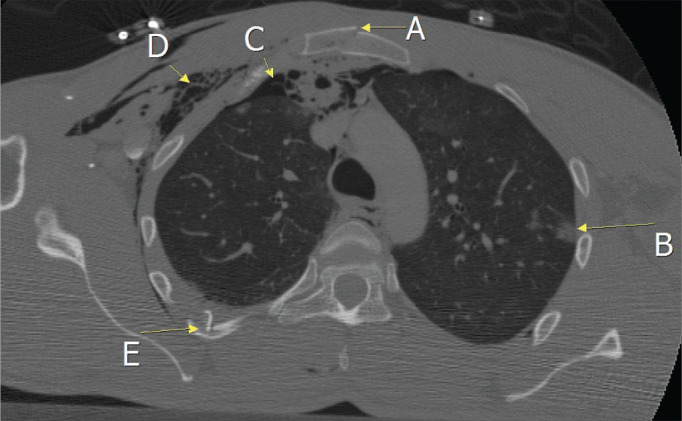
CT scan of a patient admitted because of a traffic accident, showing a fracture of the manubrium (A), pulmonary contusion (B), pneumothorax (C), subcutaneous emphysema (D), and a rib fracture (E).

In four patients (3.7%), there were no injuries to any thoracic organs. Pneumothorax was observed in 58 patients (53.7%), hemothorax was observed in 56 (51.9%), pneumomediastinum was observed in 12 (11.1%), pulmonary contusion was observed in 58 (53.7%), and pulmonary lacerations were observed in 17 (19.1%). Figure 4 illustrates the case of a patient with fracture of the manubrium accompanied by pulmonary contusion, pneumothorax, subcutaneous emphysema, and rib fracture.

**Figure 5:**
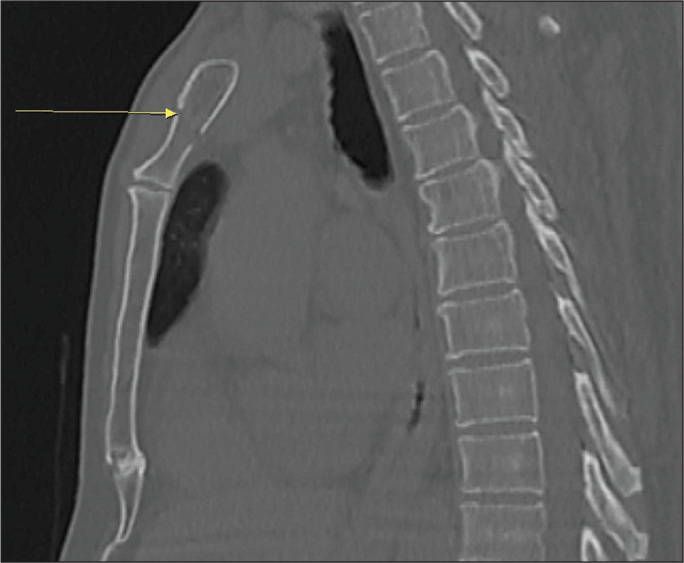
CT scan of a patient who suffered a fall from height, showing a linear fracture in the manubrium.

**Figure 6:**
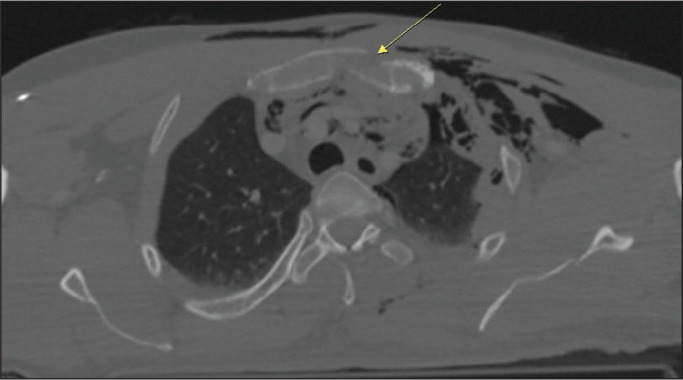
CT scan showing pneumomediastinum in a patient with a displaced fracture in the manubrium.

Fractures of the manubrium (Figure 5) were found to significantly increase the risk of pneumothorax (OR = 3.1; *p* = 0.005), hemothorax (OR = 2.3; *p* = 0.037), and pneumomediastinum (OR = 1.2; *p* = 0.004). Figure 6 illustrates the case of a patient with a displaced fracture of the manubrium accompanied by pneumomediastinum. Fracture of the sternal body was found to correlate negatively only with pneumothorax (OR = 2.63; *p* = 0.016) and pneumomediastinum (OR = 1.23; *p* = 0.003).

Among the 108 patients evaluated, intracranial hemorrhage was found in 20 (18.5%), with overlapping locations: 19 (17.6%) had subarachnoid hemorrhage; eight (7.4%) had parenchymal hemorrhage; seven (6.5%) had a subdural hematoma; and one (0.9%) had an epidural hematoma. Neither the location nor the type of sternal fracture correlated significantly with intracranial hemorrhage (*p* > 0.05 for both).

The most common solid organ injury accompanying fracture of the sternum was liver injury, which was seen in six patients (5.5%), followed by kidney injury, in one (0.9%), pancreatic injury, in one (0.9%), and adrenal injury, in one (0.9%). The patient with pancreatic injury also had duodenal bleeding. Abdominal solid organ injury did not correlate significantly with the location or type of sternal fracture (*p* > 0.05 for both).

## DISCUSSION

Of all cases of thoracic trauma, 70% are caused by blunt force and 30% result from penetrating injuries^(6)^. In a study conducted in Canada by Hill et al.^(7)^, 96.3% of the cases of thoracic trauma were reported to be blunt trauma, comparable to the 95% observed in the present study.

Thoracic trauma is most common in men between 20 and 60 years of age^(8)^. In the relevant literature, the reported proportion of males among thoracic trauma patients is 70-80%^(9)^. In the present study, the mean ages of the men and women (42.1 and 53.9 years, respectively), as well as the fact that 84.8% of our patients were male, are consistent with data in the literature showing that thoracic trauma is most common in middle-aged men who are more active in life.

The morbidity and mortality associated with fractures of the sternum are mostly attributable to concomitant injuries to internal thoracic organs, and mortality rates among affected patients range from 4% to 45%^(10)^. The mortality rate in our study was 11.1%, which is in keeping with data in the literature.

Some of the most important findings due to thoracic trauma are pulmonary contusion, hemothorax, and pneumothorax. In a study of patients with blunt thoracic trauma, conducted by Poole et al.^(11)^, the most common intrathoracic finding was pulmonary contusion, which was seen in 47% of the cases, followed by hemopneumothorax, in 27.4%. In the present study, the most common intrathoracic finding was pulmonary contusion, which was seen in 53.7% of our patients, followed by hemothorax, in 51.9%, and pneumothorax, in 53.7%.

In a significant proportion of patients, head injuries, abdominal injuries, and vertebral body fractures occur simultaneously^(11)^. In a study conducted by Başoğlu et al.^(12)^, 33.2% of thoracic trauma patients had extrathoracic injuries, 40% of which were head injuries. In another study of thoracic trauma patients, Tekinbaş et al.^(13)^ reported that there were head injuries in 11.2% and intra-abdominal injuries in 7.0%. In the present study, 10.5% of the patients had intra-abdominal injuries and 17.4% had head injuries.

In another study of patients with fractures of the sternum, Yeh et al.^(14)^ observed rib fractures at a rate of 69.9%, fractures of the scapula at a rate of 6.7%, and fractures of the clavicle at a rate of 11.3%, comparable to the 70.4%, 15.7%, and 11.3%, respectively, found in the present study, although we observed pulmonary contusions and fractures of the scapula at higher rates (53.7% vs. 29.5% and 15.7% vs. 6.7%, respectively). The authors attributed that difference to the recent increase in trauma severity.

In patients with fractures of the sternum, it is of utmost importance to identify the type and location of the fracture, because of the trauma-related findings. In a retrospective study of 272 patients with sternal fractures, Brookes et al.^(2)^ found that 93.4% had a fracture of the sternal body. In another retrospective study, von Garrel et al.^(15)^ evaluated 200 patients with sternal fractures and found that 76.5% had a fracture of the sternal body. In contrast, only 39.1% of the patients in our sample had a fracture of the sternal body. In fact, if we include the patients who had a concomitant fracture of the manubrium, that figure drops to 38.0%.

In the studies conducted by Brookes et al.^(2)^ and von Garrel et al.^(15)^, linear fractures were reported to account for 47.8% and 58.0% of sternal fractures, respectively. In the present study, linear fractures were observed in only 39.1%. In their study of patients diagnosed with fractures of the sternum between 1985 and 1991, Brookes et al.^(2)^ observed that sternal fracture was accompanied by rib fracture in 27.2% of the cases, by pneumothorax in 3.3%, by hemothorax in 5.9%, by pulmonary contusion in 3.7%, by cranial injuries in 3.7%, and by abdominal injuries in 3.3%, reporting an overall mortality rate of 0.79%. In our study, all of those rates were higher, and that difference might be attributable to the noncompliance with traffic regulations in our country and the increased trauma severity resulting from technological advances. An analysis of 1,124 victims of traffic accidents showed that the incidence of fractures of the sternum increased from 0.7% to 4.0% over a three-year period^(16)^. In addition, the use of seat belts increases the incidence of fractures of the sternum.

Despite the existence of various studies on fractures of the sternum, there has been, to our knowledge, no study regarding the relationship between sternal fracture location and injuries to other organs. In the present study, we found that fracture of the manubrium showed a statistically significant correlation with pneumomediastinum. It can be assumed that this relationship is due to fracture of the mediastinum together with the manubrium, with higher energy trauma than in a fracture of the sternal body, given the immediate proximity of the manubrium and its greater anatomical thickness and width. Similarly, rates of pneumothorax and hemothorax were found to be significantly higher among the patients with fractures of the manubrium. However, we found that the location of a sternal fracture did not correlate with intracranial injuries, abdominal injuries, or mortality.

## CONCLUSION

Blunt trauma accounts for the majority of cases of thoracic trauma, the most common causative events being traffic accidents and falls. Fractures of the sternum typically affect the manubrium and are usually linear fractures. The rate of concomitant thoracic injuries is significantly higher in patients with a fracture of the manubrium than in those with a fracture of the sternal body, which underscores the importance of correctly identifying the fracture location. In thoracic trauma with fracture of the sternum, pulmonary contusions and rib fractures are the most common intrathoracic injuries. In addition, we detected a significant positive correlation between the type of sternal fracture and the risk of lung injury; the risk of pneumothorax, hemothorax, and pneumomediastinum increases to a significant degree when the fracture is in the manubrium. Because fractures of the manubrium occur as a result of severe trauma, additional injuries attributed to the trauma increase mortality and should be thoroughly investigated.
